# Masked Syllable Priming Effects in Word and Picture Naming in Chinese

**DOI:** 10.1371/journal.pone.0046595

**Published:** 2012-10-08

**Authors:** Wenping You, Qingfang Zhang, Rinus G. Verdonschot

**Affiliations:** 1 Key Laboratory of Behavioral Science, Institute of Psychology, Chinese Academy of Sciences, Beijing, China; 2 Leiden Institute for Brain and Cognition, Leiden University Centre for Linguistics, & Cognitive Psychology Unit, Leiden University, Leiden, The Netherlands; 3 Graduate School of Languages and Cultures, Nagoya University, Nagoya, Japan; University of Leicester, United Kingdom

## Abstract

Four experiments investigated the role of the syllable in Chinese spoken word production. Chen, Chen and Ferrand (2003) reported a syllable priming effect when primes and targets shared the first syllable using a masked priming paradigm in Chinese. Our Experiment 1 was a direct replication of Chen et al.’s (2003) Experiment 3 employing CV (e.g., 拔营,/ba2.ying2/, *strike camp*) and CVG (e.g., 白首,/bai2.shou3/, *white haired*) syllable types. Experiment 2 tested the syllable priming effect using different syllable types: e.g., CV (气球,/qi4.qiu2/, *balloon*) and CVN (蜻蜓,/qing1.ting2/, *dragonfly*). Experiment 3 investigated this issue further using line drawings of common objects as targets that were preceded either by a CV (e.g., 企,/qi3/, *attempt*), or a CVN (e.g., 情,/qing2/, *affection*) prime. Experiment 4 further examined the priming effect by a comparison between CV or CVN priming and an unrelated priming condition using CV-NX (e.g., 迷你,/mi2.ni3/, *mini*) and CVN-CX (e.g., 民居,/min2.ju1/, *dwellings*) as target words. These four experiments consistently found that CV targets were named faster when preceded by CV primes than when they were preceded by CVG, CVN or unrelated primes, whereas CVG or CVN targets showed the reverse pattern. These results indicate that the priming effect critically depends on the match between the structure of the prime and that of the first syllable of the target. The effect obtained in this study was consistent across different stimuli and different tasks (word and picture naming), and provides more conclusive and consistent data regarding the role of the syllable in Chinese speech production.

## Introduction

Speaking involves conceptual preparation, lexical access, word-form encoding and articulatory processes [1]–[6]. In Levelt and colleagues’ model of language production, word-form encoding refers to the retrieval of morphemes in the mental lexicon, and is further divided into separate segmental spell-out and metrical spell-out levels [6]–[9]. Recently, the role played by sub-lexical units in this process such as the phoneme, syllable or mora has received much attention [10]–[15]. In speech perception and visual word recognition, it has been suggested that the syllable constitutes a relevant functional unit [16], [17] (see [18] for a review). However, the evidence for the functional role of syllables is still a controversial issue (see [6] for a review).

The debate about the syllable in speech production particularly addresses the question whether the syllable is stored or formed on the fly. According to Dell’s model [2], [3], the phonological syllable is a stored representation in the lexicon. During word form encoding, not only the phonological syllable units but also their metrical frames are activated mandatorily. In contrast, Levelt’s model [5], [6] assumes that word forms are not syllabified in the lexicon but computed on-line during word-form encoding. Levelt [19] proposed the existence of a mental syllabary where the syllable plays an important role at a post-phonological encoding stage [7], [20]–[24] (but see [25]).

Although many studies have been devoted to the role of the syllable in phonological word-form encoding, the evidence supporting the syllable as a functional unit is still scarce and occasionally conflicting. For instance, two studies using masked priming reported a clear syllabic priming effect in speech production [26], [27] in French and English. In Ferrand et al.’s [26] study, participants were asked to name words or pictures in French (which has a regular syllable structure and clear syllable boundaries). The target names started with a CV syllable, e.g., *pa.lace* (*palace* in English, a dot indicates a syllable boundary, similarly hereinafter), or with a CVC syllable, e.g., *pal.mier* (*palm* in English). Targets were preceded by a CV prime, e.g., *pa* or a CVC prime, e.g., *pal*, which were presented for a very short time (29 ms) to avoid conscious perception (and therefore strategic effects) by participants. They observed an interaction between the type of target and the type of prime: CV targets (e.g., pa.lace) were named faster with CV primes (pa) than with CVC primes (pal), and CVC targets (e.g., pal.mier) were named faster with CVC primes (pal) than with CV primes (pa) (for similar findings, see [28] in French and [29] in Spanish). The *syllable priming effect* supports the notion that the syllable is an essential unit during word-form encoding in speech production.

However, later studies faced difficulties in replicating the syllable priming effect (e.g. [12] in Dutch), even using identical stimuli, experimental design, and target languages in French [30] and in English [13], [14]. Instead, these studies found that the magnitude of the priming effect always increased with overlapping phoneme length between primes and targets, independent of agreement between the syllabic structure of prime and target. This effect was coined the *segmental overlap effect*. It is hard to reconcile the discrepancy between Ferrand et al.’s [26], [27] and Schiller’s [12]–[14] studies. Schiller [13] pointed out that the syllable priming effect reported by Ferrand et al. [27] for English was likely induced by methodological issues or strategic effects, such as different prime exposure duration (50 ms compared to 29 ms, see [12]; p.501). Schiller [12] (p.502) further speculated that in Ferrand et al.’s [26] crucial fifth experiment participants may have noticed that all critical items began with a CV syllable and may have used this information to strategically influence their response. Importantly, the segmental overlap hypothesis *challenged* the assumption that syllabic units are involved in word form encoding in speech production.

Chen, et al. [10] proposed that the aforementioned discrepancies might have been due to language differences. French is a syllable-timed language, whereas Dutch and English are stress-timed languages. Cutler and colleagues also offered the same explanation to account for the differences between French and English in their speech segmentation studies [31], [32]. Levelt and colleagues also suggested that language-specific differences could account for the diverging results in the speech production domain [6], [33]. In the Levelt et al. model [6], a major reason for creating phonological syllables online is the fact that words re-syllabify during online speech production (explained below). Therefore, Chen et al. [10] proposed that French might not have been the best language to test the syllable’s role during speech production as ambi-syllabicity and re-syllabification also exist in French.

Chinese, however, has quite different properties from French. There are around 400 syllables in Chinese when not counting tones and around 1,200 when counting tones (in contrast, languages such as Dutch have more than 12,000 syllables). Furthermore, syllables in Chinese are not re-syllabified in connected speech, for instance, 西安 (/xi1.an1/, *capital of Shanxi Province in China*) (a dot indicates a syllable boundary and the number indicates tone) cannot be falsely identified or pronounced as/xian1/(e.g., 鲜, *fresh*). In contrast, the syllable boundaries of a phonological word in Dutch or English differ from a lexical word’s canonical syllabification. For instance, for the word “*predict*” (*pre.dict*), different contexts require a different re-syllabification, such as “*predicted*” (*pre.dic.ted*) when using the past tense and “*predict it*” (*pre.dic.tit*) when producing the cliticization. Continuous re-syllabification in connected speech would make the storage and retrieval of syllables in the mental lexicon inefficient (except for frequently co-occurring cliticizations). Therefore, Chinese, having much less syllables and with the absence of re-syllabification, may provide an ideal testing grounds to assess the syllable’s role during speech production.

Based on the aforementioned views, Chen et al. [10] investigated the role of the syllable using a similar (masked priming) task as Ferrand et al. [26], [27]. Disyllabic Mandarin Chinese words were used as targets and single Chinese characters were used as primes. In Chen et al’s [10] third experiment, syllable overlap between the prime and the first syllable of the disyllabic target was manipulated. They found that the CV targets were named faster when preceded by CV primes compared to CVG (G represents glide sound) primes, whereas the opposite pattern was obtained for the CVG targets. The critical crossover interaction between prime type and target type was significant, and thus provides evidence for the notion that the syllable is a functional unit in speech production. In addition, studies using other production tasks such as the implicit priming task [11], [34] and picture-word interference task [35]–[37] also attested to the important role of the syllable in Chinese speech production.

However, in contrast to these results, two studies reported have found sub-syllabic priming effects in Chinese word production using a picture-word interference task, in which a target picture and a distractor word were presented simultaneously or sequentially. Wong and Chen (2008) [38] found significant facilitation effects when the target and the distractor shared the syllable, or shared the syllable plus tone. In addition, these authors observed a significant facilitation effect when the rhyme was shared (Experiment 5 using auditory distractors) or when rhyme plus tone were shared (Experiment 2 using visual distractors) between targets and distractors (see [39] for a similar finding). As different paradigms, modalities and different materials were used, this may explain the discrepancies between Wong and Chen [38], [39] and Chen et al.’s [10], [34] studies. However, Wong and Chen’s [38], [39] findings at least question the extent to which we can claim that the syllable is the fundamental unit in Chinese speech production.

Given the divergence on the role of syllable in different languages and the inconsistent findings among studies in Chinese [10], [38], [39], it is essential to investigate this issue further. Given the fact that there is currently only one study using the masked priming task in Chinese to investigate the role of the syllable [10] we propose to extend this literature by carrying out the following four experiments. Experiment 1 was designed to fully replicate the syllable priming effect reported by Chen et al. [10] using their exact stimuli and procedures. Experiment 2 aims to extend the syllable priming effect using *different syllable types* in a masked priming task. If any potential effect found by the first experiment is genuine we expect it to generalize across syllable types such as CVN (and not only for CVG).

It has been argued that the syllable priming effects may occur during input word identification in a word naming task (e.g., [12]). Previous syllabic effects found in Chinese may furthermore be due to the fact that Chinese logographic characters represent syllables (instead of individual segments as in alphabetic scripts). As Chen et al. [10] only used word naming task in their study, it is still uncertain whether these potential confounds may have played a role in their results. A task such as picture naming does not involve word identification, therefore Experiment 3 tested whether or not the masked syllable priming effect can be replicated using a picture naming task.

Chen et al. [10] directly compared syllable structure matched and mismatched conditions to examine the role of syllable, and did not include a comparison to an unrelated condition (see Chen et al. [10]; p.111 for rationale). We added this comparison by including an unrelated word prime distractor. In this way we can investigate both syllabic and segmental priming directly (without the necessity to contrast them to each other). In addition this experiment evaluates (for CVN syllables) whether or not if the overlap transcends the syllable such as a CVN prime for CV.NX target words the CV prime will be more effective (as it has sane syllabic structure with the first syllable of the target) even though the CVN prime has more overlap in terms of phonemes.

The general predictions are as follows: if the syllable priming hypothesis holds in Chinese speech production (i.e. the syllable is the functional unit to fill metrical frame), we expect to find larger facilitation for CV primes when naming CV targets (compared to CVN targets), and the reverse when CVN primes are used (i.e. larger facilitation for CVN targets compared to CV targets). If the segmental overlap hypothesis holds (i.e. the segment is the functional unit to fill metrical frame), we expect to find a larger facilitation effect whenever more segments overlap between prime and target. Our results will therefore make a substantial contribution to the debate which unit is used (syllable/phoneme) to fill the slots of the metrical frame during Chinese language production and will inform models of language production to account for a wide range of languages.

## Experiment 1: Word naming using CV and CVG targets

Experiment 1 was designed to replicate Chen et al.’s [10] findings using identical stimuli and experimental design.

### Methods

#### Participants

Seventeen undergraduate students (all female, average 23.0 years; range 21–26 years) participated and were paid approximately $3. They were randomly taken from Beijing Forestry University, China Agriculture University, and Beijing Science and Technology University. All were native Mandarin Chinese speakers with normal or corrected-to-normal vision.

#### Materials

Fifteen pairs of Chinese disyllabic words were used as targets containing CV and CVG syllable types on the first syllable (taken from Chen et al. [10], Experiment 3). For example, a pair of CV and CVG target words would be 拔营 (/ba2.ying2/, *break up camp*) and 白首 (/bai2.shou3/, *white haired*). Their corresponding primes are爸 (/ba4/, *father*) and 败 (/bai4/, *failure*), respectively. There were 30 single Chinese characters as primes, half were CV syllables, and half were CVG syllables. Each prime combined with each target word in the experiment. A neutral prime containing an asterisk sign (*) was also included.

Two CVG target words and one CVG prime character were replaced because they were unfitting in Mandarin Chinese (Chen et al. [10] used Cantonese as a target language), i.e太座 (/tai4.zuo4/, *wife*) was replaced by 太阳 (/tai4.yang2/, *sun*), 骰子(/shai3.zi5/, *dice*) was replaced by 晒干 (/shai4.gan1/, *dry*), and 譁 (/hua2/, *gorgeous*) was replaced by 滑 (/hua2/, *slippery*).

#### Design

The experiment adopted a 3 (Repetition: 1^st^, 2^nd^, 3^rd^)×2 (Target Type: CV, CVG) ×3 (Prime Type: CV, CVG, Neutral) within-participants design. Because of limited items, we manipulated the repetition exactly as Chen et al. [10] did. Participants were asked to name the target words with each prime condition three times. Each prime was accompanied by a percent sign (%) on the right side to align their positions with the targets across trials. For each pair of the target words, the following three types of primes were created: (a) CV or CVG primes that shared the first syllable with target (e.g., 爸% (/ba4/, *dad*)–拔营 (/ba2.ying2/, *strike camp*; 败% (/bai4/, *failure*)–白首 (/bai2.shou3/, *whitehaired*), (b) CV or CVG primes that matched the same segments with the initial portion of the target, but not the first syllable (e.g., 爸%,/ba4/–白首,/bai2.shou3/; 败%,/bai4/–拔营,/ba2.ying2/), (c) neutral primes that consisted of asterisk signs (e.g., *%–拔营,/ba2.ying2/; *%–白首,/bai2.shou3/). Primes and the initial characters of targets have different tones.

Each participant named 15 target pairs three times preceded by three kinds of primes, i.e., 90 trials in one repetition, three repetitions resulting in 270 trials in total. Each repetition was set in one block, resulting three blocks in total. The order of target words within a block was pseudo-randomized to prevent targets from repeating across five trials. A new sequence was generated for each participant and each block.

#### Apparatus

The experiment was performed using E-Prime Professional Software (Version 1.1; Psychology Software Tools). Participants were seated in a quiet room approximately 70 cm from a 21 inch CRT computer screen with a refresh rate of 100 Hz. Naming latencies were measured from target onset using a voice-key, connected with the computer via a PST Serial Response Box.

#### Procedure

The procedure was identical to Chen et al.’s [10] third experiment. Before the experiment, participants were told that their task was to name words. Participants first were given 20 warm-up trials in order to familiarize themselves with the experimental procedure.

Each trial involved the following sequence: a fixation cross (+) was presented at the center of the screen for 500 ms, followed by a forward mask (@@) for 500 ms, subsequently the prime was presented for 50 ms, followed by a backward mask (@@) for 20 ms. After that, the target word appeared, and disappeared after 2 seconds or when participants made a vocal response. Primes and targets were presented in 28-point boldfaced Song font, and the forward and the backward masks (@@) in 36-point Song font. The visual angles of the disyllabic target words were less than 2 degree horizontally and vertically. Participants were asked to name the word aloud as quickly and accurately as possible. Following each response, the experimenter judged whether the response was correct or not (or whether a voice key error had occurred). An inter-trial interval of 1000 ms concluded each trial. The experiment took about 20 minutes in total.

### Results

One participant was discarded from the analysis because more than 10% of his data could not be used due to a significant number of errors combined with voice key malfunctioning. For the remaining participants, incorrect responses and other responses caused by microphone errors (2.69%), RTs longer than 1000 ms and shorter than 300 ms (0.21%), and RTs deviating by more than two standard deviations from each subject’s means (4.21%) were excluded from further data analysis. The remaining data were used in subsequent statistical analyses. Table 1 presents the mean naming latencies and mean percentage of errors, presented by Prime Type and Target Type.

**Table 1:** Mean naming latencies (in ms) and mean percentage of errors (%) with standard deviation in parentheses in Experiment 1.

Primes	RT (SD)		% Error rate (SD)	
	CV target	CVG target	CV target	CVG target
CV	509 (49)	512 (49)	2.92 (2.25)	3.61 (3.96)
CVG	520 (52)	506 (47)	2.92 (3.60)	2.36 (2.50)
Neutral (*)	511 (49)	505 (44)	2.22 (2.15)	2.08 (2.06)

ANOVAs were carried out on RTs with participants (F1) items (F2) as random variables, Repetition and Prime Type as within-participants and within-items variables, and Target Type as a within-participants and between-items variable.

The main effect of repetition was significant, *F_1_*(2, 30) = 15.82, *MS_e_* = 1442.98, *p*<.001; *F_2_*(2, 56) = 166.64, *MS_e_* = 154.26, *p*<.001. Bonferroni-corrected tests indicated that target words were named faster in the second (507 ms) and the third repetition (497 ms) compared to the first repetition (527 ms) (*ps*<.05). However, importantly, repetition did not interact significantly with Prime Type or Target Type (all *Fs*≤2.29, all *ps* ≥.12). Therefore, the data were collapsed across the three repetitions.

The main effect of the Target Type was significant by participants, *F_1_*(1, 15) = 10.11, *MS_e_* = 80.78, *p*<.01, but not by items, *F_2_*(1, 28) = 0.93, *MS_e_* = 1603.15, *p* = .34. The CVG targets (508 ms) were named faster than the CV targets (513 ms). The main effect of Prime Type was marginally significant by participants, *F_1_*(2, 30) = 3.28, *MS_e_* = 56.44, *p* = .052, and significant by items, *F_2_*(2, 56) = 5.16, *MS_e_* = 79.26, *p*<.05. Bonferroni-corrected tests showed no significant pairwise comparisons (*ps* >.10). Critically, the cross-over interaction between the Target Type and the Prime Type was highly significant, *F_1_*(2, 30) = 14.17, *MS_e_* = 44.37, *p*<.001; *F_2_*(2, 56) = 12.97, *MS_e_* = 63.49, *p*<.001. Simple effects of the Prime Type, conducted for each Target Type, showed that there was a significant effect for the CV targets, *F_1_*(2, 30) = 15.57, *MS_e_* = 38.86, *p*<.001; *F_2_*(2, 56) = 14.24, *MS_e_* = 63.49, *p*<.001, and CVG targets, *F_1_*(2, 30) = 3.37, *MS_e_* = 61.95, *p*<.05; *F_2_*(2, 56) = 3.90, *MS_e_* = 63.49, *p*<.05. Bonferroni-corrected tests showed that the CV priming condition differed significantly from the CVG priming condition for all target types and the CVG priming condition differed significantly from the neutral condition for the CV target type (*p*<.05).

A similar analysis was carried out on error rates. The main effect of the repetition was significant, *F_1_*(2, 30) = 15.16, *MS_e_ = *.002, *p*<.001; *F_2_*(2, 56) = 7.97, *MS_e_* = .005, *p*<.01. Bonferroni-corrected tests indicated that the error rate was lower in the second (1.94%) and the third repetition (1.32%) compared to the first repetition (4.79%) (*ps* <.05). Furthermore, Repetition interacted with Prime Type, *F_1_*(4, 60) = 2.88, *MS_e_* = .002, *p*<.05; *F_2_*(4, 112) = 3.87, *MS_e_* = .002, *p*<.05. No other main effects or interactions were significant (all *Fs* ≤2.67, all *ps* ≥.08). Since Target Type did not significantly interact with the other two variables, the data were collapsed across Target Type in the subsequent analyses. Simple effects of Prime Type, conducted under each Repetition, showed that there was a significant effect for the first repetition, *F_1_*(2, 30) = 3.57, *MS_e_* <.01, *p*<.05, *F_2_*(2, 58) = 5.23, *MS*_e_ <.01, *p*<.01, but non-significant for both the second, *F_1_*(2, 30) = .69, *MS_e_* <.01, *p* = .51; *F_2_*(2, 58) = 1.20, *MS_e_* <0.01, *p* = .31, and the third repetition, *F_1_*(2, 30) = .21, *MS_e_* <.01, *p* = .81, *F_2_*(2, 58) = 0.24, *MS*_e_ <0.01, *p* = .79. Bonferroni-corrected tests showed that the CV priming condition (6.88%) differed significantly from the neutral priming condition (3.13%) for the first repetition (*p*<.05).

### Discussion

Experiment 1 replicated the masked syllable priming effect reported in Chen et al. [10]. The important cross-over interaction between the Prime Type and the Target Type was also observed. The CV targets were named faster when preceded by CV primes than CVG primes, and CVG targets were named faster when preceded by CVG primes than CV primes. This finding indicated that the overlapped syllable, not the pure overlap between segments, elicits greater priming, and thus supported the notion that the syllable is a functional unit during phonological encoding.

Interestingly, targets were named fastest when preceded by the neutral priming condition. Given that Chinese characters are distinctly different from the neutral primes (*%) and that neutral primes (asterisks) do not have a lexical entry, participants perhaps just discounted the perception of the neutral prime. Thus, the asterisk primes may not have been the most proper control condition. We will return to this issue in Experiment 4.

## Experiment 2 Word naming using CV and CVN targets

In Experiment 1, we replicated Chen et al. (2003) [10] using CV and CVG syllables. In Experiment 2 we will examine this further by examining whether the syllable priming effect can be replicated using other Chinese syllable structures, such as CVN syllables, which would provide additional evidence concerning the syllable as a fundamental unit during language production. In Chinese a CVN syllable type ends with/n/or/ng/(e.g.,/*xian/or/xiang/*).

### Methods

#### Participants

Seventeen undergraduate students (7 males, average 22.0 years; range 18–25 years) from the same pool as Experiment 1 participated in Experiment 2 and were paid approximately $3.0. None of them participated in Experiment 1.

#### Materials

Forty Chinese disyllabic words were chosen as targets. Half of them started with a CV syllable on the first character (CV targets), e.g., 气球 (/qi4.qiu2/, *balloon*). Half started with a CVN syllable on the first character (CVN targets), e.g., 蜻蜓 (/qing1.ting2/, *dragonfly*). Eighty single Chinese characters were selected as primes. Half were CV syllables, half were CVN syllables. For CV and CVN primes, half of them combined with CV targets, half combined with CVN targets.

Note that the CV targets and CVN targets were preceded by two different groups of characters with CV syllables and CVN syllables respectively. For instance, the primes企 (/qi3/, *attempt*) and 情 (/qing2/, *affection*) were presented before the CV target 气球 (/qi4.qiu2/, *balloon*), respectively. In contrast, the primes奇 (/qi2/, *strange*) and 顷 (/qing3/, *a little while*) were presented before the CVN target “蜻蜓” (/qing1.ting2/, *dragonfly*), respectively. Primes and the initial characters of targets have different tones. The CV and CVN targets were matched on word frequency (Chinese Linguistic Data Consortium, 2003) [40], frequency of the first character, syllable frequency of the first character (Modern Chinese Frequency Dictionary, 1986) [41], and stroke number of the first character.

Primes were chosen which did not bore any semantic nor orthographic relationship with the target words. The CV and CVN primes were matched on character frequency, syllable frequency (Modern Chinese Frequency Dictionary, 1986) [41], and stroke number. We also matched the positional probability of prime appearing at a word’s end (*Fs* <2.33, *ps* >.05) according to the type frequency and token frequency reported in Chinese Linguistic Data Consortium [40] (see also [42]). Table 2 presents the lexical properties of target words and primes by syllable type.

**Table 2:** Properties of the stimuli used in Experiment 2.

	WF	(I)CF	(I)SF	(I)SN	Probability	
					Type F	Token F
CV target	4.27	590	1093	8	–	–
CVN target	4.25	615	1112	10	–	–
Primes paired with CV target						
CV prime	–	196	838	9	38.31%	32.92%
CVN prime	–	194	834	9	54.86%	47.15%
Primes paired with CVN target						
CV prime	–	198	844	9	42.02%	44.05%
CVN prime	–	195	837	9	48.77%	51.76%

*Note:* WF = word frequency (per million); ICF = initial character frequency (per million) of target; ISF = initial syllable frequency (per million) of target; ISN = stroke number of initial character of target; CF = character frequency (per million); SN = stroke number; SF = syllable frequency (per million); Probability means the positional probability of prime appearing at a word’s end; Type F = Probability by type frequency; Token F = Probability by token frequency.

#### Design, apparatus, and procedure

Identical to Experiment 1.

### Results

Incorrect responses and other responses, e.g. caused by voice key error (2.97%), naming latencies longer than 1000 ms and shorter than 300 ms (0.23%), and those deviating by more than two standard deviations from each subject’s mean (4.40%) were excluded. The remaining data were used in the subsequent statistical analysis. Table 3 presents the mean naming latencies and the mean percentage of errors, presented by Prime Type and Target Type.

**Table 3:** Mean naming latencies (in ms) and mean percentage of errors (%) with standard deviation in parentheses in Experiment 2.

Primes	RT (SD)		% Error rate (SD)	
	CV target	CVN target	CV target	CVN target
CV	486 (39)	499 (37)	2.45 (2.37)	3.04 (2.72)
CVN	491 (36)	490 (38)	3.82 (1.75)	2.35 (2.43)
Neutral	490 (38)	494 (36)	2.75 (2.43)	3.43 (2.79)

ANOVAs were carried out on response latencies means with participants (F1) and items (F2) as random variables. We used Repetition and Prime Type as within-participants and within-items variables, and Target Type as within-participants and between-items variable.

The main effect of repetition was significant, *F_1_*(2, 32) = 40.85, *MS_e_ = *677.43, *p*<.001; *F_2_*(2, 76) = 217.15, *MS_e_* = 163.42, *p*<.001. Bonferroni-corrected tests indicated that target words were named slowest in the first repetition (510 ms), faster in the second (489 ms), and fastest in the third repetition (477 ms) (*ps* <.05). Repetition interacted with Prime Type, *F_1_*(4, 64) = 4.19, *MS_e_* = 92.01, *p*<.01; *F_2_*(4, 152) = 3.66, *MS_e_* = 142.00, *p*<.01. Simple effects of Prime Type, conducted under each Repetition, showed that there was a significant effect for the first repetition by participants, *F_1_*(2, 32) = 5.15, *MS_e_* = 54.02, *p*<.05, and marginally significant by items, *F_2_*(2, 78) = 2.85, *MS*_e_ = 243.96, *p* = .06, but insignificant for the second repetition, *F_1_*(2, 32) = .52, *MS_e_* = 56.46, *p* = .60; *F_2_*(2, 78) = .17, *MS_e_* = 186.94, *p* = .84, and for the third repetition, *F_1_*(2, 32) = 2.40, *MS_e_* = 51.54, *p* = .11, *F_2_*(2, 78) = 2.41, *MS*_e_ = 135.58, *p* = .10. Bonferroni-corrected tests showed that the CV priming condition (514 ms) differed significantly from the neutral priming condition (506 ms) for the first repetition (*p*<.05). Furthermore, the three-way interaction between Repetition, Prime Type, and Target Type repetition was not significant, *F_1_*(4, 64) = 1.53, *MS_e_ = *89.39, *p* = .21; *F_2_*(4, 152) = .66, *MS_e_* = 142.00, *p* = .62. However, importantly, the interaction between the Target Type and the Prime Type was significant, *F_1_*(2, 32) = 15.62, *MS_e_ = *83.50, *p*<.001; *F_2_*(2, 76) = 8.44, *MS_e_* = 239.35, *p*<.001. Therefore, the data were collapsed across the three repetitions for the following analysis.

The main effect of the Target Type was significant by participants, *F_1_*(1, 16) = 23.23, *MS_e_* = 33.41, *p*<.001, but not by items, *F_2_*(1, 38) = .47, *MS_e_* = 1255.55, *p* = .50. The CV targets (489 ms) were named faster than the CVN targets (495 ms). The main effect of Prime Type was not significant, *F_1_*(2, 32) = .66, *MS_e_* = 63.63, *p* = .48, *F_2_*(2, 76) = .068, *MS_e_* = 79.78, *p* = .94. As mentioned above, the cross-over interaction between the Target Type and the Prime Type was significant. Simple effects of the Prime Type, conducted for each Target Type, showed that there was a marginally significant effect by participants, *F_1_*(2, 32) = 2.70, *MS_e_* = 43.91, *p* = .08; and significant by items for the CV targets *F_2_*(2, 76) = 3.60, *MS_e_* = 79.78, *p*<.05, and a significant effect for the CVN targets, *F_1_*(2, 32) = 11.34, *MS_e_* = 30.60, *p*<.001; *F_2_*(2, 76) = 4.91, *MS_e_* = 79.78, *p*<.05. Bonferroni-corrected tests showed that the CV priming condition differed marginally (*p* = .071) from the CVN priming condition for the CV target type and that the CV priming condition differed significantly from the CVN and the neutral condition for the CVN target type. Planned comparisons revealed that the differences between the CV and the CVN priming condition were significant for all target types. For the CV targets, *t_1_*(16) = 2.50, *MS_e_* = 72.13, *p*<.05; *t*_2_(19) = 2.62, *MS_e_* = 166.98, *p*<.05, target names were produced faster when preceded by CV priming condition; for the CVG targets in opposite directions, *t_1_*(16) = 5.84, *MS_e_* = 40.04, *p*<.001; *t*_2_(19) = 3.13, *MS_e_* = 157.01, *p*<.01.

A parallel analysis was carried out on error rates. The main effect of the repetition was significant, *F_1_*(2, 32) = 7.04, *MS_e_ = *.002, *p*<.01; *F_2_*(2, 76) = 10.07, *MS_e_* = .002, *p*<.001. Bonferroni-corrected tests indicated that the error rate was lower in the third repetition (1.81%) compared to the first repetition (4.36%) (*p*<.05). The repetition variable did not interact significantly with the other two variables (all *Fs* ≤1.09, all *ps* ≥.37). Therefore, the error data were collapsed across three repetitions.

The main factors of Target Type and Prime Type did not reach significance (both *Fs* ≤0.40, both *ps* ≥.67). The Target Type by Prime Type interaction was significant by participants, *F_1_*(2, 32) = 4.91, *MS_e_* <.001, *p*<.05, but not by items, *F_2_*(2, 76) = 1.87, *MS_e_* = .001, *p* = .16. Simple effects of the Prime Type, conducted under each Target Type, showed that a significant effect for CV targets by participants, *F_1_*(2, 32) = 3.53, *MS_e_* <0.01, *p*<.05, but not by items, *F_2_*(2, 76) = 1.32, *MS*_e_ <0.01, *p* = .27, and not significant for CVN targets by participants and items, both *Fs* ≤1.50, both *ps* ≥.24. Bonferroni-corrected tests showed that the difference was marginally significant between the CV priming condition (2.45%) and the CVN priming condition (3.82%) for the CV targets (*p* = .07).

### Discussion

Similar to Experiment 1, we obtained the critical interaction between the Target Type and Prime Type in naming latencies. The results show a syllable priming effect, not the segmental overlap effect, in Chinese speech production. Specifically, although CV and CVN primes shared the same segments with CV targets, only CV primes facilitated CV targets naming compared to CVN primes. This is in line with the syllable priming hypothesis rather than the segmental overlap hypothesis. However, the priming effect for the CVN targets faces a complication as CVN primes shared more segments with CVN targets compared to CV primes; therefore, we cannot unequivocally determine whether CVN priming is syllabic or segmental. We will return to this issue in the discussion section of Experiment 3.

One possible concern is that a word naming task was employed in both Experiment 1 and 2 thereby involving linguistic processing of the input target words [7]. It has been argued that the syllable priming effects may occur during input word identification (e.g., [12]). However, a task such as picture naming does not involve word identification, therefore the following experiment tested whether or not the results of Experiment 2 could be replicated using a picture naming task.

## Experiment 3 Picture naming using CV and CVN targets

### Methods

#### Participants

Twenty undergraduate students (8 males, average 22.0 years; range 18–32 years) from the same pool as previous experiments participated in Experiment 3 and were paid approximately $3.0. None of them participated in Experiment 1 or 2.

#### Materials

Forty black-on-white line drawings were chosen as targets, picture names corresponded to the disyllabic words used in Experiment 2. The pictures were selected from a database of standardized pictures in Chinese [43]. The pictures of both CV and CVN targets were matched on name agreement, *t*(19) = 0.03, *p* = .98, with 72.55% for CV targets and 72.80% for CVN targets. The same characters with CV and CVN syllables were used as primes as in Experiment 2.

#### Procedure

Before the formal experiment, participants were asked to familiarize themselves with the target pictures by viewing each target for 2000 ms with the picture name printed below each picture. After the learning phase, participants received a picture naming test without concurrently presented names. When all pictures were named without mistakes experimental blocks were administered comprising 120 experimental trials per block. Other procedures were similar to Experiment 2.

#### Design and apparatus

Identical to Experiment 2.

### Results

Four participants were excluded because they produced more than 10% incorrect responses and also showed abundant voice key errors. Incorrect responses (3.63%), naming latencies longer than 1500 ms or shorter than 350 ms (0.17%), and those deviating by more than two standard deviations from each subject’s mean (4.53%) were excluded. Because picture naming latencies are always longer than that of word naming, we used the criteria of latencies shorter than 350 or longer than 1500 ms in picture naming to remove the outliers, a procedure similar to previous studies [12], [14]. The remaining data were used in subsequent statistical analyses. Table 4 shows the mean latencies and the mean percentage of errors, presented by Prime Type and Target Type.

**Table 4:** Mean naming latencies (in ms) and mean percentage of errors (%) with standard deviation in parentheses in Experiment 3.

Primes	RT (SD)		% Error rate (SD)	
	CV target	CVN target	CV target	CVN target
CV	600 (29)	610 (29)	3.33 (2.28)	3.75 (3.97)
CVN	613 (33)	600 (31)	4.58 (3.07)	3.33 (2.51)
Neutral	604 (36)	596 (30)	3.85 (2.08)	2.92 (2.47)

Similar ANOVAs were carried out response latency means. The main effect of repetition was significant, *F_1_*(2, 30) = 32.41, *MS_e_ = *1609.04, *p*<.001; *F_2_*(2, 76) = 195.79, *MS_e_* = 486.99, *p*<.001. Bonferroni-corrected tests indicated that target words were named faster in the second (593 ms) and the third repetition (588 ms) compared to the first repetition (631 ms) (*ps*<.05). Repetition marginally interacted with Prime Type by participants, *F_1_*(4, 60) = 2.46, *MS_e_* = 249.78, *p* = .06; but not by items, *F_2_*(4, 152) = 1.42, *MS_e_* = 339.25, *p* = .23. Bonferroni-corrected tests showed no significant pairwise comparisons (*ps* >.14). Importantly, both the three-way interaction and the interaction between repetition and Target Type were not significant (all *Fs*≤.34, all *ps* ≥.71). Therefore, the data were collapsed across the three repetitions.

The main effects of Target Type and Prime Type were not significant (all *Fs*≤2.34, all *ps*≥.10). However, the cross-over interaction between the Target Type and the Prime Type was significant, *F_1_*(2, 30) = 10.80, *MS_e_* = 100.88, *p*<.001, *F_2_*(2, 76) = 12.09, *MS_e_* = 154.49, *p*<.001. Simple effects of the Prime Type, conducted for each Target Type, showed that there was a significant effect for the CV targets, *F_1_*(2, 30) = 4.01, *MS_e_* = 156.04, *p*<.05, *F_2_*(2, 76) = 7.36, *MS_e_* = 154.49, *p*<.001, and for the CVN targets, *F_1_*(2, 30) = 8.47, *MS_e_* = 93.62, *p*<.001; *F_2_*(2, 76) = 7.07, *MS_e_* = 154.49, *p*<.01. Bonferroni-corrected tests showed that the CV priming condition differed significantly from the CVN priming condition for the CV target type (*p*<.05) and that the CV priming condition differed significantly from the CVN and the neutral condition for the CVN target type (*p*<.05).

A parallel analysis was carried out on error rates. The main effect of the repetition was significant by participants, *F_1_*(2, 30) = 5.83, *MS_e_ = *.001, *p*<.01; and marginally significant by items, *F_2_*(2, 76) = 3.06, *MS_e_* = .002, *p* = .053. Bonferroni-corrected tests indicated that the error rate was lower in the second (3.18%) and the third repetition (3.18%) compared to the first repetition (4.53%). None of the other main effects or interactions reached significance (all *Fs* ≤1.35, all *ps* ≥.25).

### Discussion

We replicated the syllable priming effect using a picture naming task. The critical interaction between the Prime Type and the Target Type was again found: naming latencies for the CV target were faster when preceded by a CV prime compared to a CVN prime, whereas naming latencies for CVN targets were faster when preceded by CVN primes compared to CV primes. Similar to Experiments 1 and 2, the latency of the neutral priming condition with signs (*%) was short compared to CV or CVN priming, an issue we will return to in Experiment 4.

Importantly, there is one possible restriction for Experiments 2 and 3: CVN primes shared more segments with CVN targets than CV primes; therefore it is not unequivocal whether the priming effect is syllabic or segmental for the CVN targets’ condition, although the results of Experiments 2 and 3 strongly point in the direction of syllable priming. Experiment 4 further examined this issue when CVN primes shared syllables or segments with targets.

## Experiment 4 Word naming using CV-NX and CVN-CX targets

This experiment further examines the question whether the previously obtained effects actually reflect syllabic priming or mainly the amount of overlap between prime and target. In this experiment we used disyllabic targets starting with either a CV or a CVN syllable. We predict that if the underlying priming mechanism is truly syllabic in nature we will acquire the most priming when the entire syllable overlaps. For instance, we predict (as in previous experiments) that for CVN-CX targets, e.g. 民居 (/min2.ju1/, *dwellings*), the prime 敏 (/min3/, *agile*) compared to 密 (/mi4/, *dense*) will show the most priming. But more importantly, for the CV-NX target 迷你 (/mi2.ni3/, *mini*) we predict that when this target is preceded by the prime 密 (/mi4/) it will also show more priming compared to when it is preceded by 敏 (/min3/) even though the CVN prime has more overlap in terms of phonemes with the CV-NX target. In other words, the potential interaction between CV primes and CVN primes in CV-NX and CVN-CX will allow us to distinguish whether the priming effect is truly syllabic or segmental in nature. In addition, instead of the asterisk (*) signs in Experiments 1–3 (which followed the exact procedure by Chen et al. [10] we replaced it by a real character/word neutral condition to better compare the magnitude of the priming effect against a control which also activates an lexical item (similar to the related primes).

### Methods

#### Participants

Twenty-five undergraduate students (6 males, average 21.8 years; range 20–26 years) from the same pool as Experiments 1–3 participated in Experiment 4 and were paid approximately $3. None of them participated in Experiments 1–3.

#### Materials

Fifteen pairs of Chinese disyllabic words were selected as targets containing CV(.NX) and CVN(.CX) syllable types on the first character. For example, a pair of CV and CVN target words would be 迷你 (/mi2.ni3/, *mini*) and 民居 (/min2.ju1/, *dwellings*). The CV and CVN targets were matched on word frequency, the frequency of the first character, syllable frequency, and the stroke number of the first character (Chinese Linguistic Data Consortium, 2003) [40]. Each target was combined with three primes, a CV prime (密,/mi4/, *dense*), a CVN prime (敏,/min3/, *agile*), and an unrelated prime (耍,/shua3/, *play*), respectively. CV and CVN Primes were chosen which did not bore any semantic nor orthographic relationship with target words. Unrelated primes had no orthographic, phonological or semantic relations with targets. The primes were matched on character frequency, syllable frequency (Chinese Linguistic Data Consortium, 2003) [40], stroke numbers, and the positional probability (see: [42]), *Fs* <1, *ps* >.40. Table 5 presents the lexical properties of target words and primes by syllable type.

**Table 5:** Properties of the stimuli used in Experiment 4.

	WF	SN	Initial character			Second character		Probability	
			CF	SF	ISN	CF	SF	Type F	Token F
Targets									
CV	1.96	16.07	393	1690	8	764	1076	–	–
CVN	1.97	17.07	407	1683	10	768	1073	–	–
Primes									
CV	–	9.20	234	1417	–	–	–	48.27%	48.11%
CVN	–	9.73	230	1423	–	–	–	42.24%	34.59%
Unrelated	–	9.67	232	1420	–	–	–	47.26%	45.56%

*Note:* WF = word frequency (per million); SN = stroke number; CF = character frequency (per million); SF = syllable frequency (per million); ISN = stroke number of initial character; Probability means the positional probability of prime appearing at a word’s end; Type F = Probability by type frequency; Token F = Probability by token frequency.

#### Design

The experiment adopted a 3 (Repetition: 1^st^, 2^nd^, 3^rd^)×2 (Target Type: CV-NX, CVN-CX)×3 (Prime Type: CV, CVN, unrelated) within-participants design. Similarly, each prime was accompanied by a percent sign (%) on the right side to align their positions with the targets across trials. For each pair of the target words, the following three types of primes were created: (a) CV or CVN primes that shared the first syllable with target, e.g., 密% (/mi4/)–迷你 (/mi2.ni3/); 敏% (/min3/)–民居 (/min2.ju1/), (b) CV or CVN primes that matched the same segments with the initial portion of the target, but not the first syllable, e.g., 敏% (/min3/)–迷你 (/mi2.ni3/); 密% (/mi4/)–民居 (/min2.ju1/), (c) unrelated primes that shared no orthography, phonology or semantic with targets, e.g., 耍% (/shua3/). Prime and the initial character of target have different tones.

#### Apparatus, and procedure

Identical to Experiment 1.

### Results

Four participants were discarded from the analysis because more than 10% of their data could not be used due to an excessive number of errors combined with voice key malfunctioning. For the remaining participants, incorrect responses and other responses, e.g. caused by unintended voice-key triggering (2.59%), naming latencies longer than 1000 ms and shorter than 300 ms (0.44%), and those deviating by more than two standard deviations from cell means from each subject’s mean (5.19%), were excluded from analysis. The remaining data were used in the subsequent statistical analyses. Table 6 presents the mean naming latencies and the mean percentage of errors, presented by Prime Type and Target Type.

**Table 6:** Mean naming latencies (in ms) and mean percentage of errors (%) with standard deviation in parentheses in Experiment 4.

	RT (SD)		% Error rate (SD)	
	CV target	CVN target	CV target	CVN target
CV prime	524 (50)	525 (49)	2.12 (2.48)	2.86 (3.07)
CVN prime	532 (46)	518 (48)	2.43 (2.52)	3.07 (2.76)
Unrelated prime	537 (48)	526 (45)	2.65 (2.50)	2.43 (2.80)

ANOVAs were carried out on response latencies with participants (F1) and items (F2) as random variables. We used Repetition and Prime Type as within-participants and within-items variables, and Target Type as within-participants and between-items variable.

The main effect of repetition was significant, *F_1_*(2, 40) = 39.28, *MS_e_ = *986.52, *p*<.001; *F_2_*(2, 56) = 156.68, *MS_e_* = 218.81, *p*<.001. Bonferroni-corrected tests indicated that target words were named slowest in the first repetition (547 ms), faster in the second repetition (522 ms), and fastest in the third repetition (514 ms) (*ps* <.05). However, importantly, repetition did not interact significantly with Prime Type or Target Type (all *Fs* ≤1.00, all *ps* ≥.37). Therefore, the data were collapsed across the three repetitions.

The main effect of the Target Type was significant by participants, *F_1_*(1, 20) = 10.13, *MS_e_* = 177.44, *p*<.01, but not by items, *F_2_*(1, 28) = 1.46, *MS_e_* = 817.03, *p* = .24. The CVN targets (523 ms) were named faster than the CV targets (531 ms). The main effect of Prime Type was significant, *F_1_*(2, 40) = 8.78, *MS_e_* = 71.23, *p*<.001, *F_2_*(2, 56) = 7.84, *MS_e_* = 63.36, *p*<.001. Bonferroni-corrected tests showed that targets were named slower in the unrelated priming condition (531ms) compared to the CV priming condition (524 ms) and CVN priming condition (525 ms) (*ps* <.05). Importantly, we obtained a significant interaction between Target Type and Prime Type, *F_1_*(2, 40) = 9.95, *MS_e_* = 62.96, *p*<.001; *F_2_*(2, 56) = 7.08, *MS_e_* = 63.36, *p*<.01. Simple effects of the Prime Type, conducted for each Target Type, showed that there was a significant effect for the CV targets, *F_1_*(2, 40) = 15.61, *MS_e_* = 56.82, *p*<.001; *F_2_*(2, 56) = 11.65, *MS_e_* = 63.36, *p*<.001, and the CVN targets, *F_1_*(2, 40) = 4. 72, *MS_e_* = 77.36, *p*<.05, *F_2_*(2, 56) = 3.27, *MS_e_* = 63.36, *p*<.05. Bonferroni-corrected tests showed that the CV priming condition differed significantly from the CVN priming condition and the unrelated priming condition for the CV target type, and that the CVN priming condition differed marginally significantly from CV priming condition (*p* = .08) and differed significantly from the unrelated priming condition for the CVN target type. The differences were not significant between the CVN priming and the unrelated priming condition (*p* = .12) for the CV targets or between the CV priming and the unrelated priming condition (*p* = 1.00) for the CVN targets.

A parallel analysis was carried out on error rates. The main effect of the repetition was marginally significant, *F_1_*(2, 40) = 2.77, *MS_e_ = *.001, *p* = .07; *F_2_*(2, 56) = 3.23, *MS_e_* = .001, *p* = .06. Bonferroni-corrected tests showed no significant pairwised comparisons (*ps* >.18). Furthermore, Repetition interacted with Prime Type, *F_1_*(4, 80) = 4.93, *MS_e_* = .001, *p*<.01; *F_2_*(4, 112) = 5.27, *MS_e_* = .001, *p*<.001. No other main effects or interactions were significant (all *Fs* ≤1.08, all *ps* ≥.35). Since Target Type did not significantly interact with the other two variables, the data were collapsed across Target Type in the subsequent analyses. Simple effects of Prime Type, conducted under each Repetition, showed that there was a marginally significant effect for the first repetition, *F_1_*(2, 40) = 3.00, *MS_e_* <.01, *p* = .06, *F_2_*(2, 58) = 2.92, *MS*_e_ <.01, *p* = .06, and insignificant for the second repetition, *F_1_*(2, 40) = .67, *MS_e_* <.01, *p* = .52; *F_2_*(2, 58) = .41, *MS_e_* <0.01, *p* = .66, but significant for the third repetition, *F_1_*(2, 40) = 5.02, *MS_e_* <.01, *p*<.05, *F_2_*(2, 58) = 5.33, *MS*_e_ <0.01, *p*<.01. Bonferroni-corrected tests showed that the CV priming condition differed significantly from the CVN priming condition for the first and third repetition (*ps* <.05). For the first repetition, more errors were made in the CV priming condition (4.29%) than in the CVN priming condition (2.06%). However, for the third repetition, fewer errors were made in the CV priming condition (0.79%) than in the CVN priming condition (3.49%).

### Discussion

We observed an interaction between Target Type and Prime Type: compared to the unrelated condition, CV primes facilitate CV targets’ naming (13 ms) reliably but did not facilitate CVN targets’ naming (1 ms), whereas CVN primes facilitate CVN targets’ naming (8 ms) but did not facilitate CV-NX targets’ naming (5 ms). The differences between the CV priming condition and the CVN priming condition were significant for both target types, that is, the largest priming effect was obtained when the entire syllable overlaps. Our findings clearly demonstrated that the CVN priming effect was not segmental but syllabic in nature.

Specifically, the syllable priming effect obtained in Experiment 4 was derived from the comparison between experimental conditions (CV and CVN primes) and unrelated condition. Naming latencies in the unrelated prime condition were significantly longer than the phonologically related prime condition. This was different from experiments 1, 2, and 3 which used asterisks as the control condition (following the experimental procedure from Chen et al. [10] and Schiller [12], [14]. Therefore, including both asterisks (Experiment 1–3) and unrelated characters (Experiment 4) improves our understanding how a diverging baselines for masked priming experiments influences naming latencies. Processing time for unrelated characters is longer (likely due to lexical activation) compared to a simple asterisk. As such, it may have provided a better baseline for the critical overlap conditions than a simple asterisk.

## General Discussion

Four experiments were reported which examined the role of the syllable during Chinese speech production. Experiment 1 was a direct replication of Chen et al.’s [10] third experiment. Experiment 2 tested the syllable priming effect using different syllable types (CV and CVN) and Experiment 3 further investigated this issue using line drawings of common objects as targets which were preceded either by a CV, CVN, or neutral primes. Lastly, Experiment 4 further examined the syllable priming effect by comparing CV or CVN syllable overlap primes vs. unrelated primes for CV-NX and CVN-CX targets. Following the syllable priming hypothesis [26], [27], an interaction between the type of prime and the type of target would indicate syllabic priming. Four experiments consistently found that CV targets were named faster when preceded by CV primes compared to when they were preceded by CVG, CVN primes or unrelated primes, whereas for CVG and CVN targets the pattern of results was reversed. These results indicate that the priming effect is most robust when there is a match between the structure of the prime (i.e. syllable) and the structure of the first syllable of the target word. This pattern is best accounted for by, at least in Chinese, adhering to the syllable priming hypothesis.

These findings corroborate with previous results obtained using a similar experimental paradigm in French [26] and English [27]. However, Schiller [13] argued that the syllable priming effect observed in French might be interpreted as a visual overlap effect between the prime and target. As there was no visual similarity between primes and targets for our Chinese stimuli, this potential confound did not exist here. Furthermore, using a picture naming task in Experiment 3, we excluded the possibility that any potential priming effect may have occurred during the perceptual and linguistic processing of the experimental stimuli. Although Experiments 1–3 directly compared CV primes and CVN primes (instead of comparing it to an unrelated prime; rationale can be found in Chen et al. [10]; p.111) to infer whether or not the priming effect was syllabic, Experiment 4 used a more conventional approach (i.e. generally used in English and Dutch studies) by comparing overlap primes with an unrelated condition and found similar results.

It is important to notice that, the syllable priming effect is found to be consistent across different tasks in Chinese. Chen et al. [34], O’Seaghdha et al. [11] and Zhang [35] used the so-called response-generation procedure (implicit priming) to investigate word-form encoding in speech production. In this task (e.g., [44], [45]), participants first learn a small set of highly associated word pairs, such as “fruit-melon”, “iron-metal”, and “grass-meadow”, prior to an experimental block. During the subsequent block they repeatedly produce the response word of each pair (“melon”) in response to the visually presented prompt word (“fruit”). The latency from the presentation of the prompt to the onset of articulation of the response is measured. Critically, the presence or absence of form overlap between the responses within a block is manipulated by making short homogenous or heterogeneous blocks of prompt-response pairs. Across the experiment, each stimulus occurs in both contexts and hence acts as its own control. All studies found a syllable priming effect or a syllable plus tone priming effect and never an onset (or segmental) priming effect. Therefore, these authors also concluded that in Chinese syllables are used to fill the metrical frame and not individual segments.

However, two studies showed inconsistent results with these and our data. Wong and Chen [38], [39] found significant facilitation effects in the rhyme related condition (using auditory and visual distractors), rhyme plus tone related condition (using visual distractors), and consonant plus vowel related condition in a picture-word interference task. Their findings indicate that segments could be a functional unit in speech production. Based on this, we would expect that greater segmental overlap would always show more priming. However, this does not agree with the findings of the present study.

There are some motivations why the current study provides additional evidence over other studies reported. First of all, due to the short prime duration (i.e., 50 ms) used in typical masked priming paradigms participants are allowed less possibilities to use strategies, in contrast to for instance picture-word interference or implicit priming tasks (e.g. noticing a rhyme relationship). Second, our findings not only consistently and repeatedly replicated previously reported data (e.g. Chen et al. [10]) it also extended the interpretation of the effects using different syllable types (i.e. CVN). In addition, Experiment 3 replicated the syllable priming effect using a picture naming task thereby excluding any possible visual word input identification confound (which could not be excluded by Chen et al.’s study). Lastly, Experiment 4 provided extra evidence for the role of syllable by showing that even when there was more overlap in terms of phonemes between prime and target (i.e. CVN primes compared to CV primes for CV.NX target) there was still greater priming when the syllable structure was similar between prime and target (i.e. CV).

The results presented in this paper were consistent across different stimuli and different tasks (word and picture naming), therefore, our study constitutes a substantial and consistent addition in favor of the syllable priming effect during Chinese speech production. However, in light of Wong and Chen’s results we acknowledge the need for further studies to assess the specific locus of these diverging results between their Cantonese and our Mandarin speech production studies.

The present results are at odds with the segmental overlap effect coined by Schiller [12]–[14]. In the literature, the segmental overlap effect was found to be reliable and consistent across various Indo-European languages such as Dutch and English and the studies which did find syllable priming such as Ferrand et al. [26], [27] could not be reliably replicated [12]–[14], [29], [30]. Given these facts, Schiller and Costa [46] suggested that the role of syllable in speech production should be reconsidered carefully, at least for Indo-European languages.

As discussed in the introduction, the properties of Chinese diverge greatly from Indo-European languages (e.g., Dutch and English), for example, re-syllabification and ambiguous syllable boundaries do not exist in Chinese. This might allow for a more dominant role of syllabic units in Chinese speech production. Evidence for this can for instance been found in speech error data (see Chen [47]), e.g. the word 清浊度 (/qing1zhuo2du4/, *clarity*) was mistakenly pronounced as/qing1du2du4/, an anticipation of the entire third syllable/du/. Using chronometrics O’Seaghdha et al. [11] directly compared the phonological encoding units between English and Chinese using a response-generation (implicit priming) paradigm. They found syllable priming effects but no onset priming effects in Chinese (Experiments 1 to 4 and 7; see also Chen and Li [48]). In contrast, they did find onset priming effect in English (Experiments 5 and 6; see also Meyer [44], [45]). Their results suggested that the unit of phonological encoding is the syllable in Chinese but the segment in English (and perhaps other Indo-European languages). In particular O’Seaghdha et al. [11] coined the term “proximate” unit. The proximate unit is stated to be the primary (or first-selected) unit during phonological encoding in production. It is also proposed that this unit may differ across languages, for instance, in Chinese the proximate unit would be the atonal syllable [11], [34]. In European language the proximate unit would constitute the phoneme (which can be adapted online during a segment-to-frame association process which fills syllabic metrical structures; see also Meyer [44], [45]; Levelt et al. [6] p. 20) and for Japanese this would constitute the mora (see [15], [49]).

Interesting comparisons can be made regarding the results of syllable priming in Chinese and in Dutch using different paradigms. Syllable priming effects were both found using masked priming task and implicit priming task in Chinese [11], [34], [35]. In contrast, syllable priming effect was found using the implicit priming task [23] but not the masked priming task (e.g., Schiller [12]) in Dutch. One reason may have been that masked priming and implicit priming tasks tap into different stages of speech production. Due to the absence of overt articulation of primes and therefore no necessary to prepare the motor programs for primes, masked priming task taps into the stage of phonological encoding [12], [26], [27]; whereas the implicit priming task taps into phonological encoding as well as phonetic encoding [23]. According to the WEAVER++ model [6], [9], syllables play a role at the interface of phonological and phonetic encoding. The abstract phonological syllables are generated and then map into phonetic syllable in spoken production. In other words: a word’s syllabification is not retrieved, but generated on the fly dependent on the context in which the word appears. Findings in Dutch using masked priming (taps into phonological encoding) and implicit priming (taps into phonological and phonetic encoding) tasks are in line with this assumption of the WEAVER++ model.

If applying WEAVER++’s assumptions to Chinese it can be inferred that a syllabary encompasses a phonetic representation having the syllable and the tone integrated together. However, the effect we found is an atonal syllable effect not a syllable plus tone effect, so it is not clear how WEAVER++ retrieves a syllable without its corresponding tone from a mental syllabary in Chinese. Therefore, we tend to agree with O’Seaghdha et al. [11] that the syllable is a stored representation (or proximate) unit and the first unit to be retrieved at the phonological encoding stage during Chinese speech production. Phonological encoding of a Chinese word involves retrieval of syllables (segmental encoding) and tones (metrical encoding) [50]. The masked syllable priming effect reflects the preparation of the syllable and facilitates the retrieval of target during phonological encoding.

It could be speculated for the word naming experiments in this study that properties of Chinese logographic characters may have been an important factor contributing to syllable priming rather than segmental overlap priming. In alphabetic languages, for instance, it is well known that words can be named on the basis of grapheme-phoneme conversion rules via a non-lexical route [51] something which is not possible in Chinese [52], [53]. In addition, Chinese logographs are syllabic in structure (i.e. every logograph represents a syllable). It might therefore be that the effects obtained are simply a matter of the type of script being used (see also Schiller [12] who stated that priming effects may arise at the stage of *input word identification*). This is another motivation why the third picture naming experiment was included in the current study as picture naming does not suffer from this potential confound. A similar concern was raised by Verdonschot et al. [15] who by using Japanese kana (a mora based script) may have introduced a bias towards the mora. However, when they changed the script to romanized Japanese (romaji) they still found essentially the same effects, leading them to conclude that for masked priming the underlying script size did most likely not play a major role in their observed effects and mora based priming was likely a property of the Japanese production system (instead of a property of the script used). Similar (future) experiments in Chinese using for instance Romanized Chinese (pinyin) may reveal whether or not script type (alphabetic vs. logographic) play a role in the observation of syllabic priming effects in Chinese.

Lastly, speech production studies usually make a distinction between naming pictures (concept to articulation) and naming printed (non-)words (orthography to articulation) as they (partly) involve different mechanisms. One can therefore speculate whether or not the involvement of syllables may also exert a different influence on picture naming compared to word naming, for instance Wong and Chen [38], [39] using picture naming found sub-syllabic effects. However, our data did not show sub-syllabic effects for picture naming therefore our pattern of results seems to rule any paradigm-specific account (see also Schiller [14] for similar results). We propose that most likely the priming effect is being located at the two common processes shared by both picture- and word naming paradigms: i.e. word-form encoding and articulation, therefore the syllable likely does plays a *similar* role in word naming and picture naming, however, additional work is needed to ascertain why Wong and Chen [38], [39] did obtain sub-syllabic priming.

To summarize, in four masked priming experiments using words (experiments 1, 2, and 4) and pictures (experiment 3) as targets, syllable priming effects, opposed to segmental priming effects, were constantly found. The present findings support the view that the syllable is a fundamental (or proximate) unit and plays an independent role at the stage of phonological encoding in Chinese speech production. We propose that the incorporation of cross-language differences will enhance speech production models’ ability to build a universal account of phonological encoding.

## Supporting Information

Appendix S1 **Stimuli used in experiment 1.**
(DOC)

Appendix S2 **Stimuli used in experiments 2 and 3.**
(DOC)

Appendix S3 **Stimuli used in experiment 4.**
(DOCX)
