# Supplementary material for: Masked Syllable Priming Effects in Word and Picture Naming in Chinese
Source: PLoS One. 2012 Oct 8;7(10):e46595. doi: 10.1371/journal.pone.0046595 (PMC3466322; doi:10.1371/journal.pone.0046595)
Supplement: Appendix S1 — Stimuli used in experiment 1. (DOC) [file pone.0046595.s001.doc]

Appendix A
Stimuli used in experiment 1
CV targets	CVG targets	CV Primes	CVG Primes	
°ÎÓª/ba2.ying2/	strike camp	°×Ê×/bai2.shou3/	whitehaired	°Ö/ba4/	father	°Ü/bai4/	fail	
ÂìÒÏ/ma3.yi3/	ant	Âòµ½/mai3.dao4/	bought	Âî/ma4/	to scold	Âõ/mai4/	stride	
ÂéÓÍ/ma2.you2/	sesame oil	Âñ·ü/mai2.fu2/	ambush	Âð/ma5/	a modal particle	Âò/mai3/	to buy	
´óÒâ/da4.yi4/	gist	´ú¹µ/dai4.gou1/	generation gap	´î/da1/	to build	´ô/dai1/	dull	
ÍØÓ¡/ta4.yin4/	make rubbings	Ì«Ñô/tai4.yang2/	sun	Ëü/ta1/	it	Ì¨/tai2/	platform	
Î÷°²/xi1.an1/	name of a city	Ï¹³¶/xia1.che3/	to waffle	Îô/xi1/	the past	Ï¼/xia2/	rosy clouds	
Õ¨Ò©/zha4.yao4/	dynamite	Õ®Îñ/zhai4.wu4/	debt	Ôú/zha1/	bundle up	Õª/zhai1/	to pick	
²èÓÍ/cha2.you2/	tea-seed oil	²ñ»ð/chai2.huo3/	firewood	²å/cha1/	plug into	²ð/chai1/	dismantle	
ÉµÑÛ/sha3.yan3/	stunned	É¹¸É/shai4.gan1/	dry in the sun	öè/sha1/	shark	É¸/shai1/	sift	
Òé°¸/yi4.an4/	bill	ÑÈÒì/ya4.yi4/	surprised	ÒÂ/yi1/	clothes	Ñ¹/ya1/	press	
Î÷Å·/xi1.ou1/	Europe	ÐÞÑø/xiu1.yang3/	cultivation	Ï¦/xi1/	sunset	Ðä/xiu4/	sleeve	
¿á°®/ku4.ai4/	craze for	¿çÈë/kua4.ru4/	step into	¿Ý/ku1/	withered	¿ä/kua1/	boast	
»¯Ñé/hua4.yan4/	lab test	»µÊÂ/huai4.shi4/	evil deed	»¬/hua2/	slippery	õ×/huai2/	ankle	
Õ£ÑÛ/zha3.yan3/	blink	Õ­Ð¡/zhai3.xiao3/	narrow	Ôü/zha1/	dregs	Õ¯/zhai4/	stockaded village	
°ÔÒµ/ba4.ye4/	superior enterprise	°ÜÀà/bai4.lei4/	scum	°Ë/ba1/	eight	°Ù/bai3/	hundred	
