# Supplementary material for: Masked Syllable Priming Effects in Word and Picture Naming in Chinese
Source: PLoS One. 2012 Oct 8;7(10):e46595. doi: 10.1371/journal.pone.0046595 (PMC3466322; doi:10.1371/journal.pone.0046595)
Supplement: Appendix S2 — Stimuli used in experiments 2 and 3. (DOC) [file pone.0046595.s002.doc]

Appendix B

Stimuli used in experiments 2 and 3

| CV and CVN targets | | CV Primes | | CVN Primes | |
| --- | --- | --- | --- | --- | --- |
| 鼻子/bi2.zi5/ | nose | 彼/bi3/ | that | 并/bing4/ | and |
| 叉子/cha1.zi5/ | fork | 岔/cha4/ | fork | 阐/chan3/ | explain |
| 车轮/che1.lun2/ | wheel | 扯/che3/ | pull | 呈/cheng2/ | present |
| 大象/da4.xiang4/ | elephant | 打/da3/ | fight | 掸/dan3/ | whisk |
| 胳膊/ge1.bo5/ | arm | 葛/ge3/ | cambric | 梗/geng3/ | peduncle |
| 吉它/ji2.ta1/ | guitar | 基/ji1/ | base | 井/jing3/ | well |
| 橘子/ju2.zi5/ | orange | 举/ju3/ | lift | 君/jun1/ | monarch |
| 蜡烛/la4.zhu2/ | candle | 垃/la1/ | garbage | 览/lan3/ | view |
| 蚂蚁/ma3.yi3/ | ant | 麻/ma2/ | hemp | 蛮/man2/ | unreasoning |
| 蜜蜂/mi4.feng1/ | bee | 弥/mi2/ | pervade | 敏/min3/ | agile |
| 耙子/pa2.zi5/ | rake | 帕/pa4/ | handkerchief | 盼/pan4/ | hope |
| 皮带/pi2.dai4/ | belt | 批/pi1/ | criticize | 聘/pin4/ | employ |
| 气球/qi4.qiu2/ | balloon | 企/qi3/ | attempt | 情/qing2/ | affection |
| 沙发/sha1.fa1/ | couch | 傻/sha3/ | stupid | 赏/shang3/ | reward |
| 梯子/ti1.zi5/ | ladder | 替/ti4/ | replace | 庭/ting2/ | courtyard |
| 袜子/wa4.zi5/ | socks | 瓦/wa3/ | tile | 往/wang3/ | to |
| 西瓜/xi1.gua1/ | watermelon | 洗/xi3/ | wash | 型/xing2/ | type |
| 牙刷/ya2.shua1/ | toothbrush | 押/ya1/ | detain | 淹/yan1/ | flood |
| 玉米/yu4.mi3/ | corn | 语/yu3/ | language | 允/yun3/ | allow |
| 栅栏/zha4.lan2/ | fence | 眨/zha3/ | blink | 章/zhang1/ | chapter |
| 斑马/ban1.ma3/ | zebra | 罢/ba4/ | stop | 扮/ban4/ | play |
| 冰箱/bing1.xiang1/ | refrigerator | 闭/bi4/ | close | 丙/bing3/ | third |
| 蛋糕/dan4.gao1/ | cake | 答/da2/ | answer | 丹/dan1/ | pill |
| 凳子/deng4.zi5/ | stool | 德/de2/ | moral | 等/deng3/ | wait |
| 钉子/ding4.zi5/ | nail | 底/di3/ | bottom | 鼎/ding3/ | vessel |
| 帆船/fan1.chuan2/ | sailboat | 法/fa3/ | law | 犯/fan4/ | offend |
| 杠铃/gang4.ling2/ | dumbbell | 嘎/ga1/ | creak | 冈/gang1/ | ridge |
| 镜子/jing4.zi5/ | mirror | 即/ji2/ | namely | 京/jing1/ | capital |
| 篮子/lan2.zi5/ | basket | 辣/la4/ | spicy | 懒/lan3/ | lazy |
| 领带/ling3.dai4/ | tie | 离/li2/ | leave | 灵/ling2/ | efficacious |
| 柠檬/ning2.meng2/ | lemon | 逆/ni4/ | backwards | 佞/ning4/ | villains |
| 盘子/pan2.zi5/ | plate | 趴/pa1/ | lie | 判/pan4/ | sentence |
| 拼图/pin1.tu2/ | puzzle | 匹/pi3/ | match | 品/pin3/ | taste |
| 蜻蜓/qing1.ting2/ | dragonfly | 奇/qi2/ | strange | 顷/qing3/ | a little while |
| 上衣/shang4.yi1/ | jacket | 啥/sha2/ | what | 商/shang1/ | business |
| 行星/xing2.xing1/ | planet | 戏/xi4/ | play | 幸/xing4/ | fortunately |
| 眼镜/yan3.jing4/ | glasses | 亚/ya4/ | Asia | 艳/yan4/ | showy |
| 樱桃/ying1.tao2/ | cherry | 仪/yi2/ | instrument | 影/ying3/ | shadow |
| 熨斗/yun4.dou3/ | iron | 迂/yu1/ | circuitous | 匀/yun2/ | uniform |
| 帐篷/zhang4.peng2/ | tent | 渣/zha1/ | residue | 掌/zhang3/ | palm |
