# Supplementary material for: Masked Syllable Priming Effects in Word and Picture Naming in Chinese
Source: PLoS One. 2012 Oct 8;7(10):e46595. doi: 10.1371/journal.pone.0046595 (PMC3466322; doi:10.1371/journal.pone.0046595)
Supplement: Appendix S3 — Stimuli used in experiment 4. (DOCX) [file pone.0046595.s003.docx]

Appendix C

Stimuli used in experiment 4

| CV targets | | CVN targets | | CV Primes | | CVN Primes | | Unrelated primes | |
| --- | --- | --- | --- | --- | --- | --- | --- | --- | --- |
| 比拟/bi3.ni3/ | metaphor | 濒临/bin1.lin2/ | be on the verge of | 闭/bi4/ | close | 殡/bin4/ | funeral | 货/huo4/ | goods |
| 茶农/cha2.nong2/ | tea farmer | 铲车/chan3.che1/ | forklift | 插/cha1/ | insert | 忏/chan4/ | repent | 非/fei1/ | non- |
| 打闹/da3.nao4/ | quarrel and fight noisily | 淡忘/dan4.wang4/ | fade from one’s memory | 达/da2/ | reach | 丹/dan1/ | pill | 维/wei2/ | dimension |
| 歌女/ge1.nv3/ | singsong girl | 跟班/gen1.ban1/ | footman | 隔/ge2/ | septa | 亘/gen4/ | stretch | 架/jia4/ | shelf |
| 济南/ji3.nan2/ | Jinan city | 近况/jin4.kuang4/ | recent situation | 积/ji1/ | amass | 筋/jin1/ | tendon | 售/shou4/ | sell |
| 历年/li4.nian2/ | over the years | 临别/lin2.bie2/ | at parting | 李/li3/ | Lee | 拎/lin1/ | carry | 淘/tao2/ | naughty |
| 玛瑙/ma3.nao3/ | agate | 满怀/man3.huai2/ | full of | 麻/ma2/ | numb | 慢/man4/ | slow | 洲/zhou1/ | continent |
| 迷你/mi2.ni3/ | mini | 民居/min2.ju1/ | dwellings | 密/mi4/ | dense | 敏/min3/ | agile | 耍/shua3/ | play |
| 皮囊/pi2.nang2/ | a man’s body | 品性/pin3.xing4/ | moral character | 坯/pi1/ | adobe | 拼/pin1/ | spell | 泰/tai4/ | peaceful |
| 气馁/qi4.nei3/ | discouragement | 秦汉/qin2.han4/ | Qin and Han dynasty | 戚/qi1/ | relative | 钦/qin1/ | admire | 烤/kao3/ | roast |
| 惹恼/re3.nao3/ | annoy | 忍让/ren3.rang4/ | tolerant | 热/re4/ | hot | 认/ren4/ | recognize | 财/cai2/ | wealth |
| 吸纳/xi1.na4/ | absorb | 欣然/xin1.ran2/ | with pleasure | 隙/xi4/ | gap | 信/xin4/ | letter | 画/hua4/ | picture |
| 衙内/ya2.nei4/ | officials as palace guards | 盐水/yan2.shui3/ | saline water | 亚/ya4/ | sub- | 眼/yan3/ | eye | 铁/tie3/ | iron |
| 益鸟/yi4.niao3/ | birds useful to agriculture | 阴雨/yin1.yu3/ | overcast and rainy | 宜/yi2/ | suitable | 隐/yin3/ | hide | 锁/suo3/ | lock |
| 杂念/za2.nian4/ | distracting thoughts | 暂定/zan4.ding4/ | tentative | 咂/za1/ | suck | 簪/zan1/ | hairpin | 街/jie1/ | street |
